# The Frailty Related Index of Comorbidities is More Strongly Associated With Length of Stay Than Other Established Measures of Frailty and Function in an Australian Subacute Inpatient Cohort

**DOI:** 10.1111/ajag.70177

**Published:** 2026-05-13

**Authors:** Rupoj Sarbaswa, Jesse Zanker, Wen Kwang Lim

**Affiliations:** ^1^ Department of Medicine and Aged Care The Royal Melbourne Hospital Melbourne Australia; ^2^ Department of Geriatric Medicine and Aged Care Western Health Melbourne Australia; ^3^ The University of Melbourne Melbourne Australia

**Keywords:** comorbidity, frailty, geriatric assessment, international classification of diseases, length of stay

## Abstract

**Objective:**

The Frailty Related Index of Comorbidities (FRIC) and Functional Independence Measure (FIM) determine activity‐based funding in Australian Geriatric Evaluation and Management (GEM) units. We examined associations between the FRIC, FIM and the Rockwood Clinical Frailty Scale (CFS) with length of stay (LOS) and discharge destination.

**Methods:**

A retrospective observational cohort study of all older adults discharged from GEM at an Australian quaternary hospital between October and December 2024 was undertaken. Demographics, the FRIC score, the FIM score and CFS were extracted from medical records. Associations with LOS and discharge to residential aged care (RAC) were examined using Spearman correlation and regression modelling.

**Results:**

Among 201 adults (mean age 82.3 ± 7.5 years), the FRIC demonstrated the strongest correlation with LOS (ρ = 0.387, *p* < 0.001) and was independently associated with LOS. Adults with longer LOS (≥ 18 days) had a higher FRIC score than those discharged earlier (6.2 vs. 3.1, *p* < 0.001). Each one‐point increase in the FRIC score (range 0 to 22.9) was associated with an additional 1.8 days of hospitalisation (*p* < 0.001). The FIM and CFS were not statistically significantly associated with LOS (*p* = 0.13 and *p* = 0.85). Higher FRIC scores were associated with increased likelihood of discharge to RAC (OR 1.15 per point increase, 95% CI 1.08–1.24, *p* < 0.001).

**Conclusions:**

The FRIC demonstrated a stronger association with LOS than FIM and CFS. The FRIC showed potential as a pragmatic marker of frailty and discharge complexity. Further prospective and multi‐centre validation is required to explore the role of the FRIC in informing LOS and activity‐based funding.

## Abstract (highlights)

**Practice Impact:**

In subacute geriatric inpatients, the Frailty Related Index of Comorbidities (FRIC) is a simple, routinely collected measure associated with length of stay and discharge complexity. The FRIC may support Geriatric Evaluation and Management (GEM) teams in early identification of patients at risk of prolonged admission, enabling earlier multidisciplinary discharge planning.

**Policy Impact:**

The findings support the incorporation of the FRIC to determine activity‐based funding for subacute geriatric care. Policy makers and leaders in health service development may explore the use of the FRIC to inform service planning, benchmarking and resource allocation, while recognising the need for further validation before broader clinical and operational application.

## 1 Introduction

### 1.1 GEM Funding in Australia

Geriatric Evaluation and Management (GEM) is a subacute inpatient service that provides comprehensive multidisciplinary care to medically stable older adults requiring ongoing rehabilitation, functional optimisation and specialist geriatric assessment prior to discharge to the community. In Australia, GEM is funded through an activity‐based funding model. The Independent Health and Aged Care Pricing Authority (IHACPA) uses the Australian National Subacute and Non‐Acute Patient (AN‐SNAP) Classification to allocate each episode of care to an end‐class, which provides an estimated length of stay and determines the corresponding level of funding [1]. Prior to July 2024, Version 4 of AN‐SNAP was used to determine GEM end‐classes based on a patient's Functional Independence Measure (FIM) [2] on admission to GEM, along with a qualifier of whether the patient had delirium or dementia [1].

In response to stakeholder feedback and growing recognition that comorbidities contribute substantially to frailty and resource utilisation, IHACPA developed Version 5 of AN‐SNAP, which was implemented in July 2024. Version 5 now incorporates the Frailty Related Index of Comorbidities (FRIC) alongside the FIM Motor to determine funding [3].

### 1.2 Origin of the FRIC

In the United Kingdom, Gilbert et al. [4] developed and validated the Hospital Frailty Risk Score, which identifies International Classification of Diseases (ICD‐10) diagnostic codes that are overrepresented among frail patients and assigns weighted scores to these diagnoses.

The Hospital Frailty Risk Score was subsequently adapted by IHACPA using the Australian Modification of ICD‐10 (ICD‐10‐AM) and validated for use in the Australian population using patient activity and cost data from 2015 to 2018 [3]. This work informed the development of the Frailty Related Index of Comorbidities (FRIC). Incorporation of the FRIC into AN‐SNAP Version 5 substantially increased the explanatory power for cost variance compared with Version 4 [3].

The FRIC is a composite measure derived from routinely collected clinical coding data, based on the presence of frailty‐associated comorbid conditions identified using ICD‐10‐AM codes [3]. The total FRIC score for an episode of GEM care is calculated as the sum of weighted scores assigned to individual diagnostic codes recorded during the admission. For example, a person with diagnoses of volume depletion (FRIC score = 2.3) and Alzheimer's dementia (FRIC score = 7.1) would have a total FRIC score of 9.4. Higher scores reflect a greater burden of frailty‐related comorbidity. The FRIC score is calculated following discharge from GEM using coded diagnostic data, which are assigned by trained clinical coders in accordance with ICD‐10‐AM coding standards, based on clinical documentation.

The FRIC has not yet been validated as a point‐of‐care clinical frailty tool, nor has it been evaluated for its role in informing real‐time clinical decision‐making or discharge planning [3]. Although the Hospital Frailty Risk Score described by Gilbert et al. [4] was associated with an increased odds of 30‐day mortality, prolonged hospital stay and 30‐day readmission, no studies to date have yet evaluated the predictive validity of the FRIC for such hospital outcomes. In contrast, more established frailty measures such as the Clinical Frailty Scale (CFS) [5] have demonstrated consistent associations between higher frailty scores and adverse hospital outcomes, including prolonged LOS, increased discharge to residential aged care (RAC) and increased mortality [5, 6].

### 1.3 Future of Frailty Measures

Frailty is a critical determinant of healthcare outcomes in older adults [5]. It is associated with increased morbidity, prolonged hospitalisation, higher mortality and greater rates of requiring transition to RAC [5, 6]. Early recognition of frailty in a patient's hospitalisation is essential for goal‐directed and individualised care planning [7].

A greater understanding of the relationship between frailty and hospital outcomes such as LOS offers several clinical and system‐level benefits [8]. Clinically, it enables anticipation of care complexity and rehabilitation needs, promotes early discharge planning and facilitates identification of patients at risk of hospital‐related complications such as deconditioning and hospital‐associated infections [6, 7]. Furthermore, improved estimation of LOS assists with optimisation of bed flow, workforce allocation and operational capacity planning within geriatric services such as GEM [9]. Importantly, estimating LOS supports clear patient and family communication which can improve patient‐centred outcomes [10].

Existing measures of frailty such as FIM are resource‐intensive and CFS can often demonstrate high inter‐rater variability, limiting their feasibility for routine use in high‐throughput analyses, particularly at a health‐system level [11, 12]. Although administrative proxies of frailty cannot substitute individualised clinical assessment, the FRIC is an example of a data‐driven approach to assessing frailty with the advantage of pragmatism, objectivity and scalability [2, 13].

### 1.4 Study Rationale and Hypothesis

Despite its incorporation into AN‐SNAP Version 5 for activity‐based funding, the FRIC has not been evaluated for its association with hospital outcomes for patients admitted to GEM. We therefore sought to examine the associations between the FRIC and key hospital outcomes, including LOS and discharge destination, and to compare its performance with the FIM and CFS. We hypothesised that higher FRIC scores would be associated with prolonged LOS and increased likelihood of discharge to RAC, and that the FRIC would demonstrate at least comparable, if not stronger, associations with these outcomes than the FIM or CFS.

Additionally, comparison of observed and estimated LOS was undertaken to assess the extent to which patient‐level LOS aligned with established AN‐SNAP benchmark LOS for each end‐class and associated funding. This provided an indication of deviation from expected cost.

## 2 Methods

### 2.1 Study Design and Setting

This was a retrospective observational cohort study conducted at the Royal Melbourne Hospital (RMH), a metropolitan quaternary hospital in Victoria, Australia. Ethics approval was granted by the Melbourne Health Human Research Ethics Committee (QA2024189). As the study used routinely collected, de‐identified clinical data, the requirement for individual patient consent was waived in accordance with institutional policies.

### 2.2 Study Period and Population

All older persons aged 65 years and above, discharged from any of the three GEM units between 1 October and 31 December 2024 were eligible for inclusion (*n* = 201). There were no exclusion criteria. The sample size was informed by a priori power calculation (see Statistical Analysis).

### 2.3 Data Collection

Data were extracted from the Royal Melbourne Hospital electronic medical records and clinical coding systems in relation to a patient's inpatient GEM admission. Demographic and admission variables included age, sex, medical record number (MRN), admission ward, admission and discharge dates, LOS and principal diagnosis.

The Frailty Related Index of Comorbidities (FRIC) score was calculated from clinical coding post‐discharge. The Functional Independence Measure (FIM) [1] was recorded by GEM allied health staff on admission to GEM. The Clinical Frailty Scale (CFS) [5] was documented in the electronic medical record by treating medical or allied health staff. Where the CFS was not recorded by the treating team (*n* = 12), it was retrospectively determined by a single geriatric medicine advanced trainee.

Estimated LOS is derived from AN‐SNAP Version 5 (2024–2025 National Efficient Price Determination 2024–2025) [14]. It represents the mean LOS for patients within a given AN‐SNAP end‐class and was used as a benchmark for comparison with observed LOS.

Level of support on admission and discharge destination was manually extracted from routine clinical documentation and then grouped for analysis (e.g., home without formal supports, home with formal supports, respite and permanent RAC) (see Table 1 in Results).

All data were de‐identified prior to analysis and stored on secure servers of the Royal Melbourne Hospital that were accessible only to authorised investigators.

### 2.4 Variables

#### 2.4.1 Outcome Variables

The primary outcome was length of stay (LOS), defined as the number of days from admission to discharge from the GEM unit. The secondary outcome was the discharge destination, which included discharge to residential aged care (RAC).

#### 2.4.2 Independent Variables

The primary independent variable was the Frailty Related Index of Comorbidities (FRIC). Comparator frailty and functional measures included the Functional Independence Measure (FIM) score (cognitive, motor and total scores) and the Clinical Frailty Scale (CFS).

#### 2.4.3 Covariates

Covariates included age, sex and level of support on admission.

### 2.5 Statistical Analysis

Based on prior observational data demonstrating a moderate association between frailty and hospital length of stay (Cohen's f^2^ = 0.15), a minimum of 89 participants were required to detect an independent association of frailty and LOS with 95% power at α = 0.05 [15]. To ensure stability of multivariable estimates and accommodate additional covariates, we targeted a sample size of at least 200 participants. Larger samples were required for analyses involving binary prolonged LOS outcomes due to limited event rates.

Descriptive statistics were used to summarise patient characteristics. Categorical variables (e.g., discharge destination) were reported as frequencies and percentages and compared using χ^2^ test.

Non‐normally distributed variables (e.g., frailty measures) were summarised using medians and interquartile ranges (IQR) and compared using Mann–Whitney *U* tests. Normally distributed variables (e.g., age) were summarised using mean and standard deviation and compared using independent‐samples *t‐*tests. For subgroup analyses, LOS was dichotomised at the cohort median to define shorter‐ and longer‐stay groups. This approach was used to facilitate comparison of clinical characteristics between groups.

Given non‐normal distributions, associations between the FRIC, FIM, CFS and LOS were assessed using Spearman's rank correlation.

Estimated and observed LOS were summarised using medians and IQRs. Differences between estimated and observed LOS were assessed using the Wilcoxon signed‐rank test. The root mean square error (RMSE) was calculated as a measure of overall deviation from expected LOS. Differences in observed LOS between estimated LOS were examined using the Kruskal–Wallis test. For analysis of agreement between observed and estimated LOS, estimated LOS values were categorised into three groups based on their distribution within the cohort (short, moderate and long estimated LOS) to explore differences in error across increasing levels of estimated LOS.

To evaluate independent associations between frailty measures and LOS, multiple linear regression modelling was performed with LOS as the dependent variable and the FRIC, FIM and CFS as independent variables. Both enter and stepwise methods were applied. Standardised β‐coefficients, *p*‐values and adjusted R^2^ values were reported. Collinearity diagnostics were used to assess multicollinearity, particularly between the FIM total and its component domains.

The association between frailty measures and discharge to RAC was examined using binary logistic regression, with discharge to RAC (yes/no) as the dependent variable. Odds ratios (OR) with 95% confidence intervals (CI) were reported. The association between the FRIC and discharge to RAC was further evaluated using receiver operating characteristic (ROC) analysis, with the Youden index used to identify the optimal cut‐off point reporting sensitivity and specificity.

All statistical analyses were performed using IBM SPSS Statistics version 30 (IBM Corp., Armonk, NY, USA) and figures generated using GraphPad Prism version 10.6.1 (GraphPad Software, San Diego, CA, USA). Statistical significance was set at *p* < 0.05, with 95% confidence intervals (CI) reported where applicable.

### 2.6 Artificial Intelligence Disclosure

ChatGPT (Version 5.2, 5.3 and 5.4) was used during manuscript preparation to assist with language editing, structural refinement and clarification of academic writing in accordance with the Australasian Journal on Ageing Author Guidelines. It was not used for generation of results or data analysis. All content was reviewed, verified and edited by the authors, who take full responsibility for the accuracy, interpretation and integrity of the work.

## 3 Results

Between 1 October 2024 and 31 December 2024, there were 201 people (65% women) discharged from GEM units with a mean age ± standard deviation of 82.3 years ±7.5 years. The median LOS was 18 days, which was used to stratify the cohort into shorter‐stay (LOS < 18 days) and longer‐stay (LOS ≥ 18 days) groups. The FRIC scores ranged between 0 and 22.9 (Table 1).

Patients with longer LOS were older with significantly higher FRIC and lower FIM scores, but not higher CFS. Among frailty measures, the FRIC demonstrated the strongest association with longer LOS, with patients in the longer‐stay group showing twice the median FRIC score compared with those discharged earlier. Lower FIM Cognition and FIM Total scores were also associated with longer LOS. In contrast, FIM Motor and CFS showed no statistically significant differences between LOS groups (Table 1).

Pre‐admission supports did not differ significantly between longer‐ and shorter‐LOS groups. However, discharge outcomes differed markedly. Patients with LOS ≥ 18 days were substantially more likely to require a higher level of support on discharge—particularly discharge to RAC (Table 1).

**TABLE 1:** Patient characteristics, stratified by median length of stay.

Characteristic	Study cohort (*n* = 201)	LOS < median of 18 days (*n* = 99)	LOS ≥ median of 18 days (*n* = 102)	*p*
Age, years, mean (SD)	82.3 (7.5)	81.3 (+/− 7.5)	83.4 (+/− 7.5)	0.04
Women, *n* (%)	130 (65)	68 (52)	62 (48)	0.24
Observed LOS, days, median [IQR]	18.0 [9.0–28.0]	—	—	
Frailty measure				
FRIC, median [IQR]	4.1 [1.8–8.3]	3.1 [0.8–5.4]	6.2 [2.8–9.9]	< 0.001
FIM Motor, median [IQR]	40 [27–53]	43 [32–55]	38.5 [26–51]	0.06
FIM Cognition, median [IQR]	21.0 [14.25–27.75]	24 [16–30]	19 [13–25]	0.004
FIM total (FIM motor + FIM cognition), median [IQR]	63.0 [47.75–78.25]	68 [50–83]	58.5 [43–74]	0.01
CFS, median [IQR]	6 [5–6]	5 [5–6]	6 [5–6]	0.33
Pre‐admission level of support				0.73
Home without formal supports, *n* (%)	101 (50)	50 (51)	51 (50)	
Home with formal supports, *n* (%)	96 (48)	48 (50)	48 (50)	
Respite, *n* (%)	1 (1)	0 (0)	1 (100)	
Permanent RAC, *n* (%)	3 (2)	1 (33)	2 (67)	
Discharge destination				< 0.001
Home without formal supports, *n* (%)	9 (4)	7 (78)	2 (22)	
Home with formal supports, *n* (%)	83 (41)	40 (48)	43 (52)	
Respite, *n* (%)	11 (6)	3 (27)	8 (73)	
Permanent RAC, *n* (%)	45 (22)	9 (20)	36 (80)	
Transfer to ED/acute hospital, *n* (%)	42 (21)	32 (76)	10 (24)	
Death during GEM, *n* (%)	6 (3)	2 (33)	4 (67)	
Transfer to inpatient psychiatry, *n* (%)	1 (1)	1 (100)	0 (0)	
Transfer to inpatient rehabilitation, *n* (%)	4 (2)	3 (75)	1 (25)	
Level of support on discharge				< 0.001
Same, *n* (%)	42 (21)	22 (52)	20 (48)	
Higher, *n* (%)	106 (53)	39 (37)	67 (63)	
Not discharged to definitive destination, *n* (%)	53 (26)	38 (72)	15 (28)	

*Note:* Not discharged to a definitive destination includes ED or acute hospital transfer, death during GEM admission, inpatient psychiatry transfer or inpatient rehabilitation transfer.

Abbreviations: CFS, Clinical Frailty Scale; ED, emergency department; FIM, Functional Independence Measure; FRIC, Frailty Related Index of Comorbidities; GEM, Geriatric Evaluation and Management; IQR, interquartile range; LOS, length of stay; RAC, residential aged care.

**FIGURE 1:**
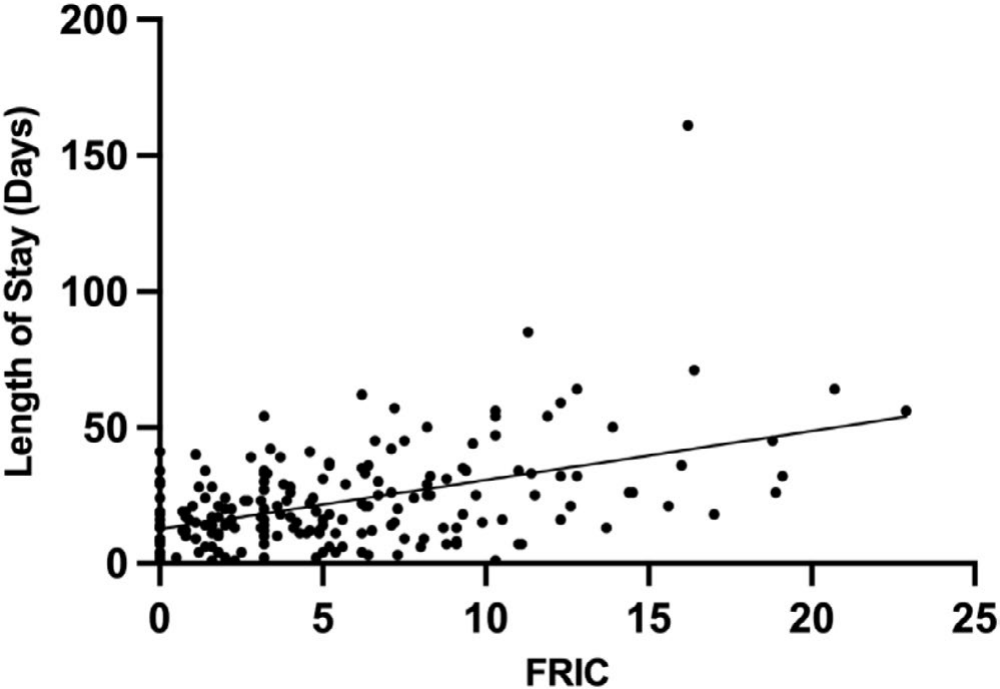
Association between Frailty Related Index of Comorbidities (FRIC) and observed length of stay. Each data point represents an individual admission. The solid line represents the fitted linear regression (R^2^ = 0.2, *p* < 0.001).

There was a moderate, statistically significant, positive association between FRIC scores and observed LOS (Spearman's ρ = 0.387, *p* < 0.001). In linear regression analysis, higher FRIC scores were associated with longer admissions, explaining 22% of the variance in LOS (R^2^ = 0.221). Each one‐point increase in the FRIC score was associated with an increase of 1.8 days in LOS (β = 1.804, *p* < 0.001) (Figure 1).

The median observed LOS in this cohort was 2.6 days lower than estimated LOS derived from the IHACPA's National Efficient Price Determination 2024–2025 [14]. There was a weak positive association between estimated and observed LOS (Spearman's ρ = 0.178, *p* < 0.01). Estimated LOS weakly overestimated observed LOS; however, this difference was not statistically significant (Z = −0.638, *p* = 0.52). The deviation from expected LOS was substantial with a Root Mean Squared Error (RMSE) of 9.4 days and was highly right‐skewed (skewness 12.3). When stratified by estimated LOS category, absolute error increased with longer estimated length of stay (Kruskal–Wallis H = 7.6, *p* = 0.034) (Table 2).

**TABLE 2:** Agreement between observed and estimated length of stay.

Measure	Result
Observed LOS, median [IQR], days	18.0 [8.5–27.5]
Estimated LOS, median [IQR], days	20.6 [18.5–22.8]
Absolute difference in LOS, median [IQR], days	
Short stays (≤ 17.1 days) (*n* = 82)	7.9 [2.4–13.4]
Moderate stays (> 17.1 to ≤ 20.6 days) (*n* = 66)	8.6 [3.4–14.0]
Long stays (> 20.6 days) (*n* = 53)	11.6 [4.5–18.9]

*Note:* Short, moderate and long stay categories refer to estimated LOS.

Abbreviations: IQR, interquartile range; LOS, length of stay.

Compared to the FIM and CFS, the FRIC scores demonstrated the strongest correlation with observed LOS. FIM‐Motor demonstrated a weak but statistically significant correlation with LOS, whereas correlations for FIM‐Total and FIM‐Cognition were weak and non statistically significant. The CFS did not show meaningful correlation with LOS. In multivariable linear regression analysis, the FRIC score was the only variable independently associated with LOS, with each one‐point increase in the FRIC score resulting in a 1.8 day increase in LOS. Neither the FIM Total score nor CFS were independently associated with LOS (Table 3).

**TABLE 3:** Comparison of frailty measures in relation to length of stay.

Frailty measure	Spearman's ρ (*p*)	β coefficient (*p*)
FRIC	+0.387 (< 0.001)	1.769 (< 0.001)
FIM‐motor	−0.080 (0.03)	—
FIM‐cognition	−0.134 (0.06)	—
FIM total	−0.106 (0.13)	−0.038 (0.496)
CFS	+0.013 (0.85)	−0.160 (0.86)

Abbreviations: CFS, Clinical Frailty Scale; FIM, Functional Independence Measure; FRIC, Frailty Related Index of Comorbidities.

Multivariable linear regression modelling demonstrated that the FRIC independently explained 22% of the variance in LOS (Figure 1). Although statistically significant, models incorporating frailty measures such as the FIM and CFS in addition to the FRIC increased R^2^ by 0.006 or less. Overall, frailty models that excluded the FRIC demonstrated poor explanatory performance (Table 4).

**TABLE 4:** Explanatory power of frailty models for observed length of stay.

Frailty model	R^2^	∆R^2^ vs. FRIC‐only	*p*
FRIC	0.217	—	< 0.001
FRIC + FIM‐Total	0.222	+0.005	< 0.001
FRIC + FIM‐motor	0.223	+0.006	< 0.001
FRIC + FIM‐cognition	0.221	+0.004	< 0.001
FRIC + CFS	0.221	+0.004	< 0.001
FIM‐total + CFS	0.020	−0.197	0.14
FIM‐motor + CFS	0.009	−0.208	0.40
FIM‐cognition + CFS	0.030	−0.187	0.05
FRIC + FIM‐motor + CFS	0.223	+0.006	< 0.001
FRIC + FIM‐cognition + CFS	0.221	+0.004	< 0.001
FRIC + FIM‐total + CFS	0.223	+0.006	< 0.001

Abbreviations: CFS, Clinical Frailty Scale; FIM, Functional Independence Measure; FRIC, Frailty‐Related Index of Comorbidities.

**FIGURE 2:**
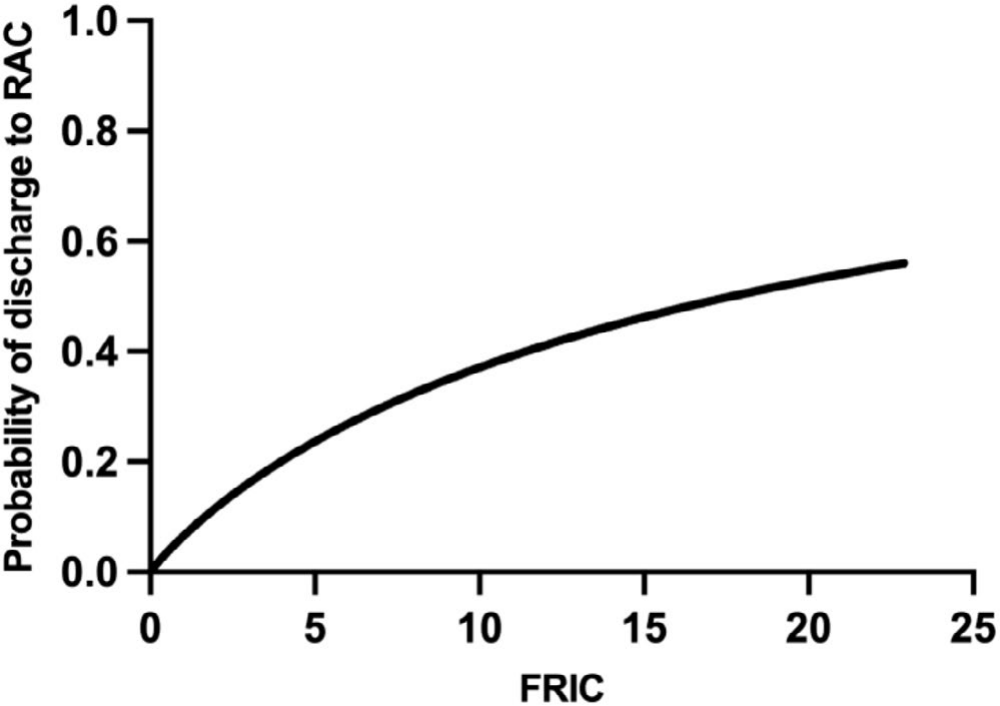
Predicted probability of discharge to residential aged care (RAC) by Frailty Related Index of Comorbidities (FRIC). The curve represents predictions from a logistic regression model.

Binary logistic regression modelling shows that higher FRIC scores were independently associated with discharge to RAC (*p* < 0.001). Each one‐point increase in the FRIC score was associated with a 15% increase in odds of discharge to RAC (OR 1.15, 95% CI 1.08–1.24, *p* < 0.001). Receiver operating characteristic analysis (ROC) identified an optimal FRIC threshold of ≥ 5.5 using the Youden index (0.31), yielding a sensitivity of 63% and specificity of 68% for identifying patients discharged to RAC (Figure 2). Furthermore, at this threshold (FRIC ≥ 5.5), the positive predictive value (PPV) is 37% and the negative predictive value (NPV) is 86% (Table S1, Table S2).

## 4 Discussion

### 4.1 Key Findings

In this retrospective cohort study of GEM inpatient admissions, increasing FRIC score was associated with greater LOS (Figure 1). The FRIC demonstrated the strongest association with LOS compared to the FIM and CFS. Compared to the FIM and CFS, the FRIC score was the only variable independently associated with LOS in multivariable regression analyses (Table 3). The addition of the FIM and CFS were not statistically significantly associated with LOS and did not contribute meaningful incremental explanatory power when added to FRIC‐based frailty models (Table 4).

Substantial variability was shown between observed LOS and LOS estimated by AN‐SNAP Version 5 (2024–2025 National Efficient Price Determination 2024–2025) [14]. Although there was no evidence of systematic over‐ or under‐estimation at a cohort level, deviations were large and greater with longer admissions, highlighting the inherent complexity of LOS determination in older inpatients living with frailty (Table 2).

The FRIC was also independently associated with discharge to RAC, with each one‐point increase in the FRIC score corresponding to a 15% increase in the odds of discharge to RAC (Figure 2). Notably, at the optimal threshold (≥ 5.5), FRIC demonstrated a high negative predictive value (86%), suggesting potential utility in identifying patients at lower risk of discharge to RAC within this cohort (Table S1).

To our knowledge, this is the first study to examine the relationship between the FRIC, hospital LOS and discharge outcomes in a GEM population.

### 4.2 Interpretation

These findings support the conceptual premise underpinning the inclusion of the FRIC within AN‐SNAP Version 5—that cumulative comorbidity burden is a key driver of hospital resource use in older adults in GEM. Unlike function‐based measures (e.g., FIM), which may fluctuate during admission and are influenced by rehabilitation processes, the FRIC captures pre‐existing disease burden that likely constrains recovery trajectories, complicates discharge planning and increases vulnerability to hospital‐associated complications [4].

Importantly, the overall modest R^2^ values observed across all comparative models (Table 4) indicate that LOS is strongly influenced by multiple determinants beyond frailty alone, including social complexity, availability of community services, organisational processes, system‐level constraints and patient and family preferences [16].

Substantial discrepancy was observed between observed LOS and LOS estimated by AN‐SNAP Version 5, especially as LOS increases (e.g., above 20.6 days) (Table 2). In this current model of activity‐based funding, the system potentially underestimates the cost of prolonged admissions.

### 4.3 Limitations

This was a single‐centre study conducted in a metropolitan quaternary GEM service, which may limit generalisability to other settings including regional or rural hospitals with differing patient populations, staffing models, admission criteria and access to community services. This may have introduced selection bias, as quaternary GEM units may over‐represent patients with higher comorbidity burden and clinical complexity, which could inflate the observed association between frailty and LOS. Conversely, metropolitan GEM units may have shorter LOS than rural centres given potentially greater access to community supports and discharge destinations such as RAC facilities [17]. The overall magnitude of this bias is likely modest, but its direction may differ depending on the comparator setting.

The FRIC is derived retrospectively from ICD‐10‐AM diagnostic coding data following discharge from GEM and therefore is dependent on local coding guidelines, accuracy, coder interpretation and completeness. This introduces potential measurement bias, with under‐ or overrepresentation of specific diagnoses. Furthermore, although recurrent episodes of delirium during the same admission potentially reflected greater clinical frailty and greater LOS, ICD‐10 coding guidelines only record a single episode of delirium—which in turn underestimates the FRIC.

Comparative frailty measures such as the FIM were typically recorded at GEM and CFS was recorded at variable times during the GEM admission, whereas the FRIC was derived retrospectively at discharge. The difference in timing introduces temporal misclassification, as functional status may change substantially over the course of a GEM admission, while comorbidity burden captured by the FRIC tends to be stable. As a result, admission FIM and CFS may not fully reflect frailty status across the admission period, potentially complicating their observed associations with LOS. This temporal mismatch further limits the direct comparability of functional and comorbidity‐based frailty measures in explaining LOS. Moreover, FIM and CFS were obtained from routine clinical documentation rather than standardised assessments protocolised for research. Inter‐rater variability and timing of assessment (particularly for CFS) may have introduced further measurement error [11]. This may lead to under‐ or overrepresentation of CFS or FIM, impairing their comparison with the FRIC.

Additionally, a small number of CFS data (12/201) were recorded retrospectively based on review of electronic medical records by a single data collector. Although this may have introduced interrater variability or measurement bias, the small number involved is unlikely to have materially influenced the results.

Finally, although regression modelling was used to examine associations, this study was not designed to develop or validate a clinical prediction model, and no external validation or out‐of‐sample testing was performed. However, given the conservative framing of results as explanatory rather than predictive, this limitation is unlikely to materially affect the principal conclusions.

### 4.4 Future Directions

The clinical and operational utility of the FRIC is currently limited by its late availability in the patient journey, as it is derived post‐discharge. Prospective studies are therefore needed to evaluate whether the FRIC can be operationalised earlier during admission. Artificial‐Intelligence‐assisted clinical coding and integration with electronic health records may advance its role in the clinical care of older inpatients [18, 19]. Earlier availability in the admission course, such as during the acute hospital phase prior to transfer to GEM, may facilitate timely recognition of frailty burden, supporting early, pathway‐led discharge planning, improved patient communication and more efficient resource allocation.

The Electronic Frailty Index (eFI) [20], most widely used in the National Health Service (NHS, UK) general practice setting, is an example of an electronic medical record‐mediated high‐throughput frailty measure. Use of the eFI has been associated with clinical benefits, including anticipatory care planning and triggering of comprehensive geriatric assessment, as well as system‐level benefits such as population risk stratification, resource allocation and service planning [20]. Similar to the eFI, the FRIC has the potential to function as an automated, routinely extractable risk stratification tool within subacute and other inpatient settings.

To date, the wider application of the FRIC is also constrained by a lack of local validation studies examining its prospective use. However, overseas studies conducted in acute hospital populations in France and South Korea have demonstrated that electronically derived frailty indices based on diagnostic codes are associated with clinically relevant geriatric syndromes when applied early during admission, supporting the feasibility of early, high‐throughput frailty assessment in hospital settings [21, 22].

Finally, given the substantial influence of non‐comorbidity‐related factors on LOS, further research is required to explore integration of the FRIC with psychosocial, environmental and system‐level variables to more comprehensively explain the variation in hospital outcomes including LOS in older inpatients.

## 5 Conclusions

In this study of subacute geriatric inpatients, the FRIC demonstrated an independent association with hospital LOS and discharge destination, outperforming established frailty measures such as the FIM and CFS. Higher FRIC scores were linked to longer admissions and increased likelihood of discharge to RAC, indicating that the FRIC captures the impact of chronic comorbidity burden on subacute hospital resource utilisation.

Although developed as a funding and cost‐classification tool, the FRIC shows promise as an example of a pragmatic, system‐level marker of clinical frailty and indicator of level of support required on discharge from subacute hospital care.

Future research is encouraged to evaluate the prospective utility of the FRIC, validate its performance across diverse inpatient geriatric care settings, and examine integration with functional and psychosocial variables to enhance its clinical and operational applications.

## Funding

Jesse Zanker is supported by a National Health and Medical Research Council of Australia Emerging Leadership 1 (EL‐1) Investigator Grant (2042450).

## Ethics Statement

Ethics approval was granted by the Melbourne Health Human Research Ethics Committee (QA2024189). As the study used routinely collected, de‐identified clinical data, the requirement for individual patient consent was waived in accordance with institutional policies.

## Conflicts of Interest

The authors declare no conflicts of interest.

## Supporting information


**Table S1:** Receiver operating characteristic analysis of the FRIC for prediction of discharge to residential aged care.
**Table S2:** Operating characteristics of the FRIC across selected thresholds.

## Data Availability

The data that support the findings of this study are available from the corresponding author upon reasonable request.
