# Supplementary material for: The Frailty Related Index of Comorbidities is More Strongly Associated With Length of Stay Than Other Established Measures of Frailty and Function in an Australian Subacute Inpatient Cohort
Source: Australas J Ageing. 2026 May 13;45:e70177. doi: 10.1111/ajag.70177 (PMC13170412; doi:10.1111/ajag.70177)
Supplement: Supplementary file 1 — Table S1: Receiver operating characteristic analysis of the FRIC for prediction of discharge to residential aged care. Table S2: Operating characteristics of the FRIC across selected thresholds. [file AJAG-45-0-s001.docx]

**Supplementary Table S1:** Receiver operating characteristic analysis of the FRIC for prediction of discharge to residential aged care

| **Measure** | **Value** | **95% CI** | ***p*** |
| --- | --- | --- | --- |
| Area Under the Curve (AUC) | 0.695 | 0.608 - 0.782 | < 0.001 |
| Optimal Cut-off (Youden Index) | ≥5.5 (0.31) | - | - |
| Sensitivity | 0.630 | - | - |
| Specificity | 0.684 | - | - |
| Positive Predictive Value (PPV) | 0.372 | - | - |
| Negative Predictive Value (NPV) | 0.862 | - | - |

*CI = confidence interval

**Supplementary Table S2:** Operating characteristics of the FRIC across selected thresholds

| **FRIC Threshold** | **Sensitivity** | **Specificity** | **PPV** | **NPV** | **Youden Index** |
| --- | --- | --- | --- | --- | --- |
| ≥ 4.0 | 0.70 | 0.55 | 0.30 | 0.86 | 0.25 |
| ≥ 5.0 | 0.65 | 0.63 | 0.33 | 0.85 | 0.28 |
| ≥ 5.5 | 0.63 | 0.68 | 0.37 | 0.86 | 0.31 |
| ≥ 6.5 | 0.52 | 0.75 | 0.38 | 0.84 | 0.27 |
| ≥ 7.5 | 0.41 | 0.79 | 0.39 | 0.83 | 0.20 |

*Abbreviations: FRIC = Frailty-Related Index of Comorbidities; PPV = positive predictive value; NPV = negative predictive value
